# Frozen elephant trunk procedure for complex aortic arch surgery: The Salerno experience with Thoraflex hybrid

**DOI:** 10.1111/jocs.16086

**Published:** 2021-10-18

**Authors:** Paolo Masiello, Generoso Mastrogiovanni, Oreste Presutto, Pierpaolo Chivasso, Vito Domenico Bruno, Mario Colombino, Mario Miele, Francesco Cafarelli, Rocco Leone, Donato Triggiani, Severino Iesu

**Affiliations:** ^1^ Department of Emergency Cardiac Surgery, Cardio‐Thoracic‐Vascular University Hospital “San Giovanni di Dio e Ruggi D'Aragona” Salerno Italy; ^2^ Department of Translational Health Bristol Medical School Bristol UK

**Keywords:** aorta and great vessels, clinical review

## Abstract

**Background and Aim of the Study:**

To report early clinical outcomes of the frozen elephant trunk (FET) technique for the treatment of complex aortic diseases after transition from conventional elephant trunk.

**Methods:**

A single‐center, retrospective study of patients who underwent hybrid aortic arch and FET repair for aortic arch and/or proximal descending aortic aneurysms, acute and chronic Stanford type A aortic dissection with arch and/or proximal descending involvement, Stanford type B acute and chronic aortic dissections with retrograde aortic arch involvement.

**Results:**

Between December 2017 and May 2020, 70 consecutive patients (62.7 ± 10.6 years, 59 male) were treated: 41 (58.6%) for emergent conditions and 29 (41.4%) for elective. Technical success was 100%. In‐hospital mortality was 14.2% (*n* = 12, 17.1% emergent vs. 10.3% elective, *P* = NS); 2 (2.9%) major strokes; 1 (1.4%) spinal cord injury. Mean follow‐up was 12.5 months (interquartile range, 3.7–22.3). Overall survival at 3, 6, 12, and 24 months was 90% (95% confidence interval [CI], 83.2—97.3), 85.6% (95% CI, 77.7–94.3), 79.1% (95% CI, 69.9–89.5), 75.6% (95% CI, 65.8–86.9) and 73.5% (95% CI, 63.3–85.3). There were no aortic re‐interventions and no distal stent graft‐induced new entry (dSINE); 5 patients with residual type B dissection underwent TEVAR completion.

**Conclusions:**

In a real‐world setting, FET with Thoraflex Hybrid demonstrated feasibility and good clinical outcomes, even in emergent setting. Our implant technique optimize cerebral perfusion reporting good results in terms of neurological complications. Techniques to perfect the procedure and to reduce remaining risks, and consensus on considerations such as standardized cerebral protection need to be reported.

**Abbreviations**
AAD: acute aortic disease
AKI: acute kidney injury
CAD: chronic aortic disease
CO: cardiac output
CPB: cardiopulmonary bypass
CSF: cerebrospinal fluid
CT: computed tomography
CVA: cerebrovascular accident
CVVH: continuous veno‐venous hemofiltartion
FET: frozen elephant trunk
LCA: left carotid artery
LSA: left subclavianarterty
mRS: modified Rankin scale
SCI: spinal cord ischemia
TEE: transesophageal echocardiography

## 1 INTRODUCTION

The frozen elephant trunk (FET) procedure has become established as a proven and attractive option to treat aortic disease when the arch and the thoracic aorta are involved to facilitate the conventional two‐stage access.^1^ In acute and chronic aortic dissection, the use of FET can help to expand and stabilize the true lumen and cover eventual supplementary tears but the perceived technical complexity of the operation may be restricting its adoption, especially in the acute setting.^2^, ^3^, ^4^ Undoubtedly, the constant development of new branched prostheses, which increases the surgeons' armamentarium in the treatment of complex aortic arch pathology, plays an important role in reducing the risk of procedural failure.^5^ At present, there is little evidence weighing the burden of replacing the aortic arch as an additional procedure during elective or emergency proximal aortic repair, thus making comparison with patients undergoing secondary total arch replacement difficult.^6^ The aim of this study was to evaluate safety and short‐term outcomes after FET with the Thoraflex™ Hybrid (Terumo Aortic) prosthesis in aortic arch reconstruction both in emergency and elective setting at a single institution.

## 2 METHODS

This is a single‐center, retrospective, observational study based on prospectively collected data obtained from institutional cardiac surgery data set at University Hospital San Giovanni di Dio and Ruggi d'Aragona in Salerno, Italy. The study was conducted in accordance with the principles of the Declaration of Helsinki. Institutional board approval was obtained for the study, and patient consent was waived.

All patients who underwent FET for acute and chronic arch and thoracic aorta pathologies between December 2017 and May 2020 were included. The dates were chosen to capture all routine use of the Thoraflex Hybrid FET device which consists of a proximal unstented tubular gelatin‐coated Dacron graft and a distal stent‐graft polyester made with a self‐expandable nitinol skeleton, deployable antegrade during circulatory arrest over a guidewire. We use exclusively the Plexus configuration with four integrated lateral branches: three for the reconstruction of supra‐aortic vessels and one for systemic perfusion.

We size the stent‐graft portion according to the aortic diameter of the distal landing zone as evaluated by preoperative CT angiogram: 0% oversizing in acute and chronic aortic dissections; 10%–15% oversizing in ascending aorta and/or arch aneurysms, particularly when a second stage was anticipated. To minimize the risk of spinal cord ischemia, we only implant the 100 mm length.

### 2.1 Surgical technique

**Figure 1:**
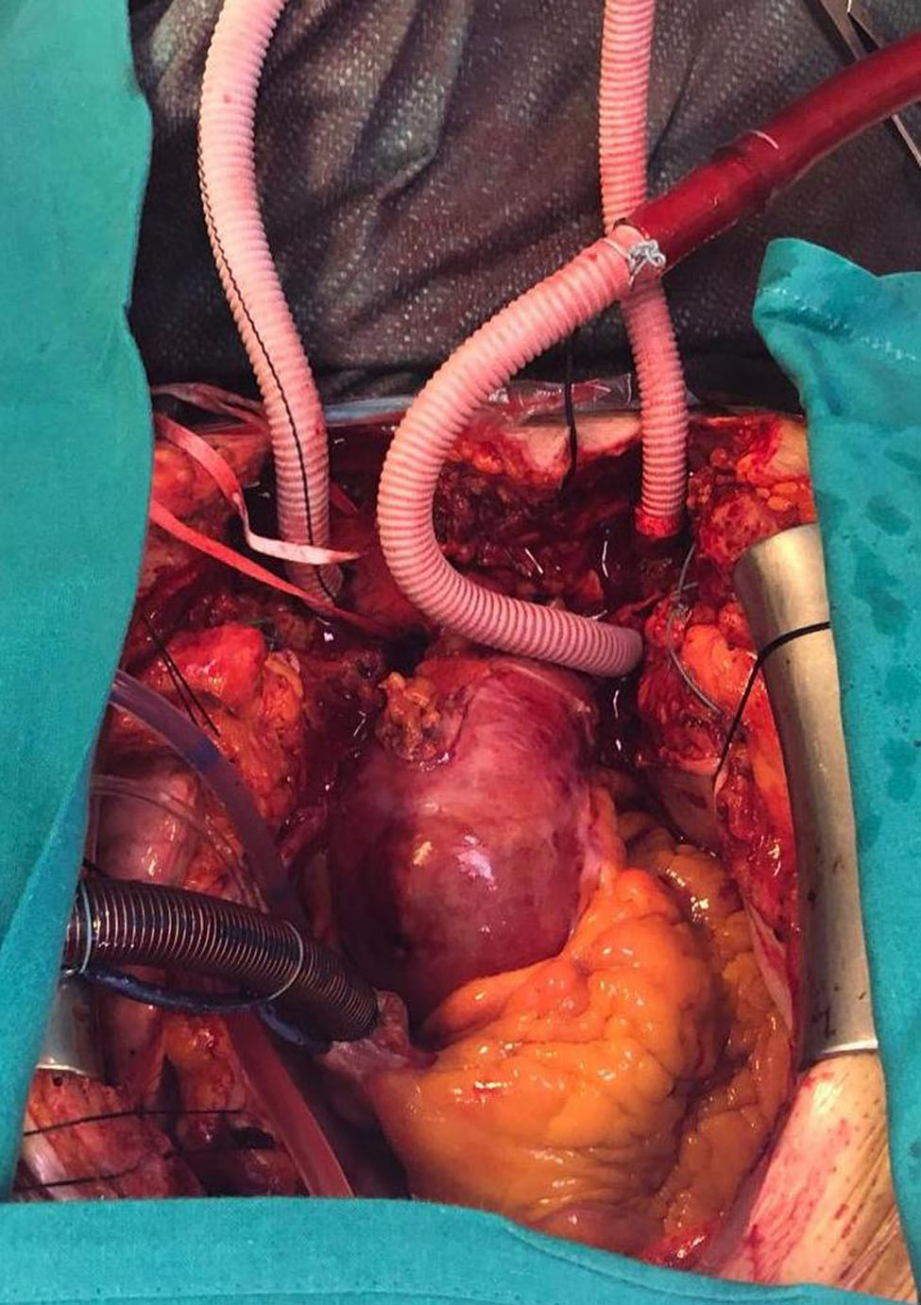
Selective cannulation of left carotid artery (LCA) and the left subclavian artery (LSA) with interposition of a Dacron graft

All cases are operated under general anesthesia after invasive arterial pressure monitoring of bilateral radial arteries and a femoral artery. We cannulate the right jugular vein after oro‐tracheal intubation and position a Swan‐Ganz catheter. All patients are monitored with a continuous transesophageal echocardiography (TEE) and bilateral cerebral oxymetry (INVOS™ system). Median sternotomy is performed in all cases: the usual incision is extended in a small right or bilateral supra‐clavicular cervicotomy to improve access and harvesting of supra‐aortic vessels. Central cannulation is routinely via the right intrathoracic subclavian artery via side graft. Our approach is “branch‐first” and beating heart arch vessel reconstruction. During the initial cooling phase, on a beating heart, the left carotid artery (LCA) and the left subclavian artery (LSA) are isolated and prepared for selective cannulation with the interposition of a 8–10 mm Dacron graft (respectively for LCA and LSA) to avoid direct cannulation of the artery (Figure 1). The vessel perfusion is sequentially started to achieve complete antegrade cerebral perfusion. Bladder and esophageal temperature are monitored. Right atrium and right superior pulmonary vein are cannulated for venous return and venting.

After debranching completion at 30°C core temperature, the aorta is cross‐clamped and opened and a single dose of Custodiol® cardioplegia is administered for cardiac protection. Proximal aortic reconstruction varies according to underlying pathology. Patients are cooled to 26°C for hypothermic circulatory arrest and the brachiocephalic artery clamped and selective antegrade perfusion begun at 10–12 ml/kg/min, consequently stopping systemic circulation. INVOS is monitored and radial pressure maintained at 60–80 mmHg. The aortic arch is then opened and inspected.

The landing zone (usually zone 2) is reinforced with Teflon strip and, eventually, bioglue. At this stage, the distal stent‐graft of the FET device is released into the descending thoracic aorta. The reinforced collar of the prosthesis is sutured to the aorta and, after cannulation of the fourth lateral branch and careful de‐airing, systemic perfusion is resumed, starting to rewarm the body. The anastomosis between the surgical graft and sinotubular junction (either native or prosthetic, depending on the proximal repair) is completed and cross‐clamp released allowing the heart to start beating. The prosthesis‐elongated supra‐aortic vessels are then end‐to‐end sequentially re‐anastomosed to the corresponding branches of the graft, starting with the LSA to LCA and finally brachiocephalic artery. The correct deployment and fully expansion of the prosthesis is assessed by TEE.

### 2.2 Cerebral and systemic perfusion

**Figure 2:**
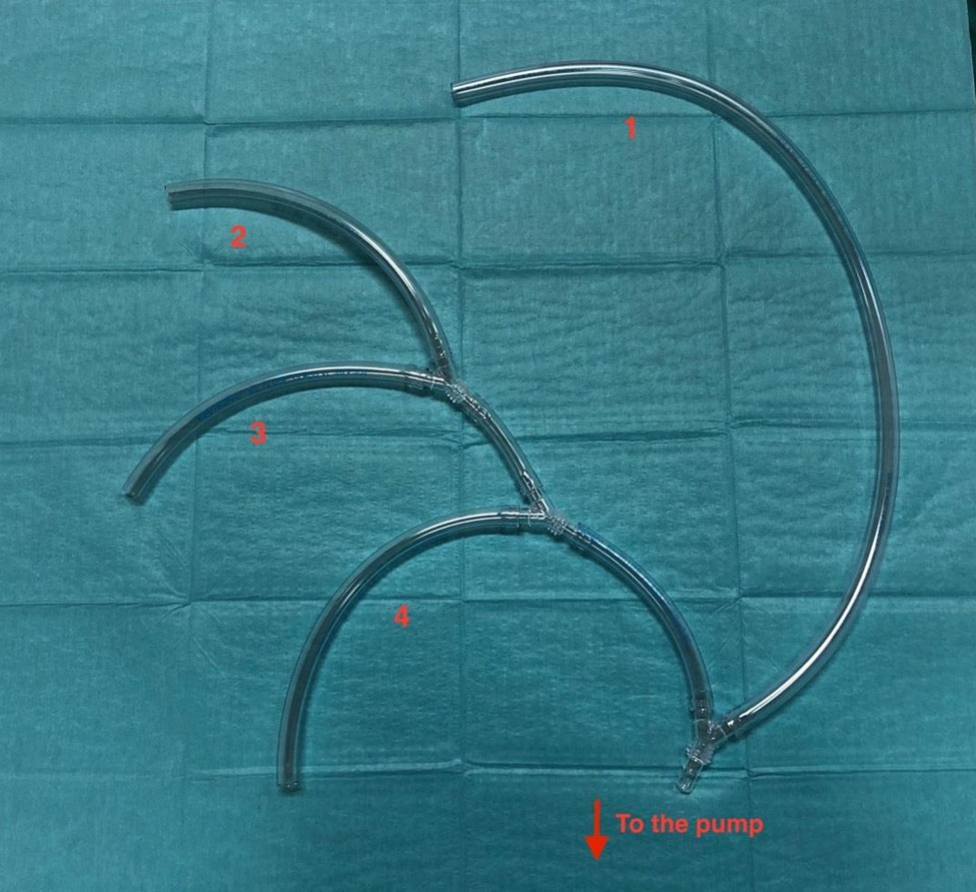
Homemade perfusion system driven by a single pump: 1, right intrathoracic subclavian artery perfusion (systemic perfusion); 2, left subclavian artery perfusion; 3, left carotid artery perfusion; 4, side branch systemic perfusion

Extracorporeal circulation and cerebral antegrade perfusion are performed using a homemade, 4‐branched perfusion circuit (Figure 2). It consists of four branches of the same diameter driven by a single pump LSA, LCA, brachiocephalic artery, and prosthetic branch for systemic perfusion. Perfusion is kept at full flow for CPB and it redistributes depending on physiological systemic resistance. When the brachiocephalic artery is clamped, the flow is lowered to 10–12 ml/kg/min for isolated cerebral perfusion. After the circulatory arrest time the full flow was restarted via prosthetic side branch and single cerebral vessels.

### 2.3 Endpoints

The primary endpoints of the study were 30‐day and in‐hospital mortality, defined as death due to any cause during postoperative course at 30 days and until discharge, usually to a rehabilitation unit, respectively. Secondary endpoints included postoperative major stroke (defined as clinical and radiological evidence of a new postoperative cerebrovascular event [CVA] with a mRS ≥4), spinal cord injury (SCI) temporary or permanent, return to operating room for cardiac causes, renal failure requiring replacement therapy, respiratory insufficiency requiring prolonged ventilation and/or tracheostomy, deep sternal wound infection involving sternal bone and/or mediastinal structures, recurrent laryngeal nerve palsy and in‐hospital length of stay.

### 2.4 Statistical analysis

Data are presented as mean and standard deviation (SD) if numerical and as count and percentages if categorical. Normality was assessed using Shapiro–Wilk test. Continuous numerical variables have been compared using the Student *t* test, while categorical variables have been compared with *Χ* ^2^ or Fisher exact test as appropriate. Time to event analysis has been conducted using Log‐rank test and displayed with Kaplan–Meier curves. Univariable and Multiple Cox proportional Hazard models were run to identify potential factors affecting midterm survival with hazard ratios (HR) and confidence intervals (CI). The final multivariable model was obtained using those variables that had a significant *p* value at the univariable analysis with backward/forward selection based on AIC. Alpha error was set at 0.05 for significance and all tests are two‐sided. The statistical analysis was conducted with R version 3.6.0. R: A language and environment for statistical computing. R Foundation for Statistical Computing, Vienna, Austria. URL https://www.R-project.org/).

## 3 RESULTS

Between December 2017 and May 2020, 70 consecutive patients (mean age 62.7 ± 10.6 years, 84% male) underwent FET for arch and thoracic aorta pathologies, 41 (58.6%) acute aortic dissections (AAD) and 29 (41.6%) chronic pathologies: ascending aorta/aortic arch aneurysm (An) in 19 (24.3%) and chronic type I dissection (CAD) in 10 (14.2%). The distributions of baseline characteristics for the overall population are presented in Table 1. Twelve (17.1%) patients had already undergone cardiac surgery. Fifteen AAD patients (36.5%) presented with peripheral malperfusion. There were no significant differences in terms of age, gender, and other preoperative comorbidities between the groups. The only significant difference was previous cardiac surgery (redo) (4.9% in emergency group vs. 34.5% in elective group, *p* < .01) Patients presenting in emergency setting had a higher incidence of malperfusion but this difference did not reach statistical significance.

**Table 1:** Preoperative characteristics

Characteristics	Overall	Emergency	Elective	*p* value
	*N* = 70	*n* = 41	*n* = 29	
Age (years)	62.76 (10.6)	61.80 (9.7)	64.10 (11.9)	.38
Male gender	59 (84.3)	35 (85.4)	24 (82.8)	1
COPD	17 (24.3)	9 (22.0)	8 (27.6)	.796
History of hypertension	65 (92.9)	38 (92.7)	27 (93.1)	1
CKD	5 (7.1)	3 (7.3)	2 (6.9)	1
History of cancer	6 (8.6)	2 (4.9)	4 (13.8)	.379
Peripheral vascular disease	11 (15.7)	5 (12.2)	6 (20.7)	.53
Previous cardiac surgery	12 (17.1)	2 (4.9)	10 (34.5)	.004
Left ventricle ejection fraction (%)	58.11 (6.18)	57.44 (7.26)	59.07 (4.16)	.28
Cerebral malperfusion at presentation	4 (5.7)	4 (9.8)	0 (0)	na
Abdominal malperfusion at presentation	5 (7.1)	5 (12.2)	0 (0)	na
Lower limb ischemia at presentation	6 (8.6)	6 (14.6)	0 (0)	na
Ascending aorta/arch aneurysm	19 (24.3)	0 (0)	19 (65.5)	<.001
Chronic aortic dissection	10 (14.2)	0 (0)	10 (31)	.001
De Bakey dissection classification				<.001
I	44 (62.8)	38 (92.7)	6 (20.6)	
III B	7 (10.0)	3 (7.3)	4 (13.7)	
Stanford dissection classification				<.001
A	42 (61.8)	38 (92.7)	4 (14.8)	
B	9 (12.8)	3 (7.31)	6 (20.6)	

*Note*: Data are reported as mean (SD) for numerical variables and as count (%) for categorical variables.

Abbreviations: COPD, chronic obstructive pulmonary disease; CKD, chronic kidney disease.

No intraoperative deaths were recorded. Operative characteristics and their distributions among the two groups are showed in Table 2. Concomitant procedures were required in 36 (51.4%) patients: 12 (17.1%) coronary artery bypass grafting, 10 (14.3%) aortic valve replacement, 14 aortic root surgery such as 9 (12.9%) Florida sleeve procedures and 5 (7.1%) modified Bentall. There was a statistically significant difference between the two groups in terms of cardiopulmonary bypass time (212.9 ± 42.7 min in the emergency group vs. 175.5 ± 51.1 min in the elective group, *p* < .01) and aortic cross‐clamp time (123.8 ± 38.9 min in the emergency group vs. 87.1 ± 31.9 in the elective group, *p* < .01). The mean deep hypothermic circulatory arrest time was 30.6 ± 6 min and did not differ among the two groups.

**Table 2:** Operative carachteristics

	Overall	Emergency	Elective	*p* value
	*N* = 70	*n* = 41	*n* = 29	
CPB time (min)	197.46 (49.49)	212.95 (42.47)	175.55 (51.08)	.001
Aortic cross‐clamp time (min)	108.64 (40.29)	123.85 (38.89)	87.14 (31.97)	<.001
HCA time (min)	30.61 (6.80)	29.63 (6.06)	32.00 (7.63)	.153
Concomitant procedures				
CABG	12 (17.1)	8 (19.5)	4 (13.8)	.762
AV Replacement	10 (14.3)	4 (9.8)	6 (20.7)	.347
Aortic root surgery				
Florida Sleeve	9 (12.9)	8 (19.5)	1 (3.4)	.106
Modified Bentall	5 (7.1)	5 (12.2)	0 (0.0)	.139

*Note*: Data are reported as mean (SD) for numerical variables and as count (%) for categorical variables.

Abbreviations: AV, aortic valve; CABG, coronary artery bypass graft; CPB, cardio‐pulmonary bypass; HCA, hypotermic circulatory arrest (including selective antegrade cerebral and visceral perfusioin).

Postoperative outcomes are summarized in Table 3. Thirty‐day mortality was 10.0% (*n* = 7) for the entire cohort of patients (12.1% in emergency vs. 6.8% in chronic settings, *p* = .421). Cumulative in‐hospital mortality was 14.2% (*n* = 12, 17.1% in emergency vs. 10.3% in chronic settings, *p* = .312). No differences were found in terms of postoperative CVA (mRS 5/6, 2.4% in the emergency group vs. 0% in the elective group, *P* = 1), SCI with paraplegia (2.4% in the emergency group vs. 0% in the elective group, *P* = 1) and AKI requiring hemodialysis (31.7% in the emergency group vs. 20.7% in the elective group, *p* = .454). The incidence of respiratory failure requiring tracheostomy did not differ between the two groups (31.7% vs. 24.1%, *p* = .673).

**Table 3:** Postoperative outcomes

	Overall	Emergency	Elective	*p* value
	*N* = 70	*n* = 41	*n* = 29	
30‐day mortality	7 (10.0)	5 (12.1)	2(6.8)	.421
In‐hospital mortality	10 (14.3)	7 (17.1)	3 (10.3)	.312
Prolonged ventilation	23 (32.9)	15 (36.6)	8 (27.6)	.595
ICU duration (days)	15.77 (16.6)	16.95 (17.9)	14.10 (14.7)	.485
Hospitalization (days)	32.11 (21.6)	31.56 (23.7)	32.90 (18.5)	.801
Return to operating room	8 (11.4)	6 (14.6)	2 (6.9)	.535
Low CO syndrome	32 (45.7)	21 (51.2)	11 (37.9)	.392
Respiratory failure	42 (60.0)	25 (61.0)	17 (58.6)	1
Tracheostomy	20 (28.6)	13 (31.7)	7 (24.1)	.673
Anemia	55 (78.6)	32 (78.0)	23 (79.3)	1
Pericardial effusion requiring drainage	16 (22.9)	12 (29.3)	4 (13.8)	.219
Pleural effusion requiring drainage	15 (21.4)	12 (29.3)	3 (10.3)	.108
Deep sternal wound infection	7 (10.0)	6 (14.6)	1 (3.4)	.258
Recurrent laryngeal nerve palsy	8 (11.4)	6 (14.6)	2 (6.9)	.535
AKI requiring CVVH	19 (27.1)	13 (31.7)	6 (20.7)	.454
Spinal cord injury/paraplegia	1 (1.4)	1 (2.4)	0 (0.0)	na
Permanent CVA	1 (1.4)	1 (2.4)	0 (0.0)	na
Lower limb ischemia	1 (1.4)	1 (2.4)	0 (0.0)	na

*Note*: Data are reported as mean (SD) for numerical variables and as count (%) for categorical variables

Abbreviations: AKI, acute kidney injury; CO, cardiac output; CVVH, continuous veno‐venous haemofiltartion; CVA, cerebrovascular accident; ICU, intensive care unit.

The final multivariable Cox proportional hazard model, reported in Table 4, showed that higher LVEF (HR, 0.93; 95% CI, 0.88–0.99; *p* = .03) was a protective factor for midterm survival, while cerebral malperfusion at presentation (HR, 4.42; 95% CI, 1.19–16.33; *p* = .03) and aortic cross‐clamp time (HR, 1.01; 95% CI, 1–1.02; *p* = .05) were found to be independent predictor factors affecting midterm survival. Emergency surgery did not significantly impact on survival (elective HR, 0.60; 95% CI, 0.21–1.7; *p* = .33).

**Table 4:** Univariable and multivariable Cox proportional hazard model

Variables	HR
Age, years	1.05
Gender, Male	0.38
LVEF (perc.)	0.94
COPD	0.97
PVD	2.40
History of CAD	1.40
Previous cardiac surgery	0.62
Cerebral malperfusion	3.74
Abdominal malperfusion	2.75
Peripheral malperfusion	2.43
CPB time (min)	1.01
Cross clamp time (min)	1.01
Cerebral perfusion time (min)	0.98
Elective surgery	0.60

*Note*: Data are reported as mean (SD) for numerical variables and as count (%) for categorical variables.

Abbreviations: AMI, acute myocardial infarction; CI, confidence intervals; CPB, cardio‐pulmonary bypass; eGFR, effective glomerular filtration rate; HR, hazard ratio; LVEF, left ventricular ejection fraction.

There were no aortic re‐interventions in either group. Five patients with residual type B dissection underwent TEVAR successful completion of repair which was performed at least 6 weeks after the primary operation to reduce the spinal cord ischemia risk. There was no dSINE and no intraluminal thrombosis.

**Figure 3:**
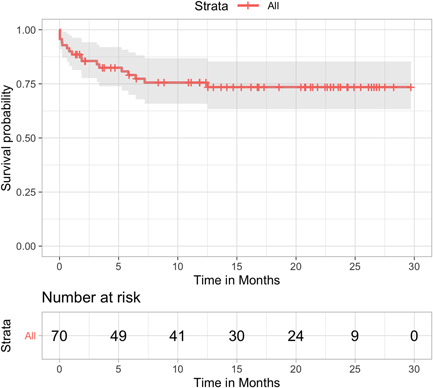
Kaplan–Meier survival curve for the overall surgical population

**Figure 4:**
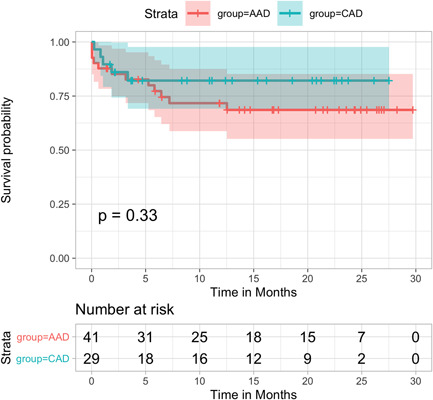
Kaplan–Meier survival curves between the two groups (raw data). AAD, acute aortic disease treated in emergency setting; CAD, chronic aortic disease treated in elective setting

Median follow‐up was 12.5 months (interquartile range, 3.9–22.3). Overall survival for the entire cohort at 3, 6, 12, and 24 months was 90% (95% CI, 83.2–97.3), 85.6% (95% CI, 77.7–94.3), 79.1% (95% CI, 69.9–89.5), 75.6% (95% CI, 65.8–86.9) and 73.5 (95% CI, 63.3–85.3), respectively (Figure 3). Survival rate by group were 85.2% versus 86.1% at 3 months, 77.3% versus 82.2% at 6 months, 71.7% versus 82.2% at 12 months, and 68.6% versus 82.2% at 24 months, all emergency versus elective, respectively (Figure 4).

## 4 DISCUSSION

Surgical repair of aneurysms and aortic dissections involving the aortic arch is still challenging, carrying high mortality and morbidity. Our experience with the first 70 consecutive patients treated with FET using Thoraflex Hybrid has been overall satisfactory; technical success was achieved in all patients even in the first part of our experience, started after performing only conventional ET or conventional surgery in acute dissection (ascending aorta or hemiarch replacement). Our results show that the implantation technique for Thoraflex Hybrid graft is doable and reproducible, also in emergent setting.

Correct cerebral perfusion during hypothermic circulatory arrest is one of the main factors determining neurological outcomes.^7^, ^8^, ^9^ Our strategy allows a uniform cerebral perfusion throughout the operation, except for the short time needed for LCA and LSA anastomosis, thus minimizing cerebral ischemia time. We believe that this technical aspect is important to explain our particularly good results in terms of neurological complications, with a significantly lower overall incidence of permanent stroke (1.4%) compared to data from similar studies.^7^ Shresta et al. reported 8% of major CVA in their first experience in performing a FET with the Thoraflex with a different brain protection strategy, while Chu et al. reported a 5% stroke incidence.^10^, ^11^ In addition to our cerebral perfusion strategy, we believe that our technique, avoiding direct cannulation and manipulation of cerebral vessels, could reduce the embolism rate thus helping to minimize CVA complications.

Another interesting finding from our study is the very low rate of spinal cord injury which occurred in only one patient (1.4%) operated for acute aortic dissection. We believe that our good results are due to the use of a combination of 100 mm stented length (less intercostal arteries coverage), Thoraflex Hybrid deployment in zone 2, short circulatory arrest time, and correct sizing of the stented graft.^12^ Fiorentino et al. reported a low overall incidence of SCI (two cases of temporary isolated papaparesis) but only in 150 mm distal stented grafts.^13^ Flores et al reported a very high incidence of SCI in FET when the stented was deployed at the lower level of the thoracic aorta.^14^

It has been reported that in Thoraflex implant the LSA anastomosis remains the Achilles heel being too close to the collar device.^11^ We overcome this problem by extending the surgical incision with a small left supra‐clavicular cervicotomy and “elongating” the LSA with a tubular prosthesis, thus making the anastomosis technically easier and achieving success in all cases. In one case, not included in this series, we successfully used a custom‐made Thoraflex Hybrid, in which the plexus is separated from the main part of the prosthesis, to make anastomosis easier, improve operating times and correctly position the intra‐thoracic vessels.^15^

Overall survival for the entire cohort at 30 days was 90% (95% CI, 83.2–97.3), without statistically significant differences between emergency and elective surgery (87.9% vs. in emergency vs. 93.2% in chronic settings, *p* = .312).

We believe that our results should contribute to encourage employing this surgical strategy, especially in emergency setting where the FET may be helpful particularly in patients with malperfusion and could result in a definitive treatment. The idea of specialized centers with a high volume of aortic surgery to treat both chronic and acute aortic syndrome is now becoming paramount. There is a large consensus that patients affected by acute aortic syndromes may benefit from treatment at dedicated specialized aortic centers with significantly improved outcomes and decreased mortality. Patients undergoing emergency repair of acute aortic dissection by lower‐volume surgeons and centers have approximately double the risk‐adjusted mortality of patients undergoing repair by the highest volume care providers.^16^ We think that the future treatment of acute type A aortic dissection is going toward a total arch approach with standardized cerebral protection that should more and more be delivered by specialist aortic centers with expertize in this technique. In this case, the Thoraflex Hybrid proved to be an easy‐to‐implant prosthesis, making the brain protection strategy easier and reporting a low complication rate.

Finally, an important aspect of our study is the relatively large number of cases done in a short period of time in a single institution, thus allowing for a significant reduction of multicenter studies bias. Other series available in literature report results of a similar cohort of patients but operated in different centers: the Canadian experience enrolled 40 consecutive cases in 9 different centers, in about 3 years of activity while the English experience counts 66 cases in 4 years from 9 centers throughout UK.^17^

### 4.1 STUDY LIMITATIONS

Due to the relatively low complication rate and limited follow‐up, it was not possible to detect differences between the groups or pathologies. At present, the follow‐up has a shorter duration compared to other series.

## 5 CONCLUSIONS

Surgical techniques involving stenting of the descending thoracic aorta during primary surgery for both acute and chronic complex aortic disease involving the arch are associated with promising early and midterm results. Result of this study and growing evidence in the literature suggest that FET in acute aortic dissection should also be routine when performed by experienced operators in dedicated aortic centers.

## CONFLICT OF INTERESTS

The authors declare that there are no conflict of interests.

## AUTHOR CONTRIBUTIONS

*Concept/Design*: Masiello, Mastrogiovanni. *Data analysis*: Bruno, Presutto. *Drafting article*: Masiello, Chivasso, Cafarelli. *Critical revision*: Masiello, Mastrogiovanni, Iesu. *Approval of article*: Iesu. St*atistical*: Bruno. *Data collection*: Presutto, Leone, Triggiani, Colombino, Miele.
